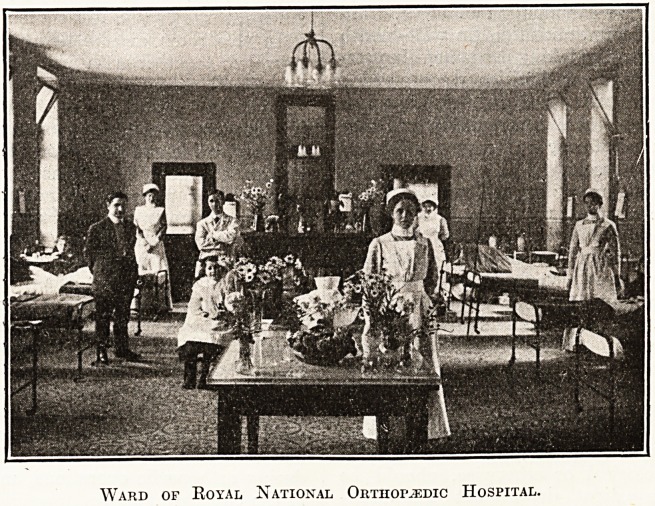# The Modern Orthopædic Unit

**Published:** 1913-12-13

**Authors:** 


### THE ORTHOPÆDIC DEPARTMENT.*

### IV.—The Modern Orthopædic Unit (*continued*).

### THE OUT-PATIENT DIVISION.

The out-patient division of the unit should form part of the general out-patient department of the hospital, but should, at the same time, be so arranged that it is virtually a separate unit of that department. With the structural questions involved we do not propose to deal here; they belong, more particularly, to the general subject of the planning of the out-patient department which we propose to discuss elsewhere. All that need be said is that in this unit, as in all the others, the best work can only be obtained under the best conditions and with the best means. Cheapness here is false and vicious economy, which ultimately must defeat its own object. We are fully aware that there is a tendency to ignore these considerations and to maintain that the success of all work depends on the men and not on the means or the environment. That, of course, is a fact, but it is a fact which has certain unavoidable limitations, so that it is a bad policy for hospital constructors to hold it in view to the exclusion of other considerations. Nothing can be more earnestly impressed upon the minds of all concerned in hospital work than the fact that initial structural mistakes and the provision of cheap and inefficient material instead of durable, if slightly more expensive, material mean finally far more expense in renovation and restoration than would have been entailed had, in the first instance, the best material and the most correct planning been the rule. With these remarks as preface we may proceed to deal with the out-patient division.

This should consist of three main rooms, and should be within easy access of the other two divisions, and more especially of the third or treatment department. In fact, the latter is usually incorporated in this division, but for purposes of description it is more convenient to differentiate them in the manner we have done here. Access to the ward is of less importance since patients destined for in-treatment are never emergency cases, but usually chronic cases, which can be removed to the ward at leisure. This fact makes it unnecessary for the out-patient division to have an emergency ward room such as, for instance, must be provided on the surgical side. The out-patient department of the unit is primarily an examination and consultation centre. Here the orthopædic surgeon first sees his cases, and treats those that he can on the spot or refers them to the ward or treatment division respectively. The absolute requirements of the division, so far as the out-patient block is concerned, are, therefore, relatively easy to satisfy. As a minimum we may reduce them to three essentials, namely, an examination room with a room for the staff, and a waiting room attached, to which, of course, proper sanitary annexes should be planned; an operating room for minor cases that demand operative treatment on short notice (even this may be dispensed with or incorporated with the third essential), and, thirdly, a dressing room.

Ward of Royal National Orthopædic Hospital.

The examining room should be roomy and comfortable, and furnished with a table, an examining couch, a weighing machine, and a measuring apparatus, a screen, a glass cupboard for diagnostic apparatus (see below), chairs for the surgeon, his assistant or clerk, and for the patient, a small cupboard (with lock) for reagents and certain drugs, a shelf for disinfectants, and an adjustable electric

* Previous articles appeared on November 1, 15, and 29.

lamp. Hot and cold water taps with suitable washing basins should be provided, and it is an advantage to have a backed window blind which enables the room to be darkened for examination of the eye or larynx. In addition to the usual writing materials, patients' letters and case sheets, certain instruments must be provided for diagnostic purposes. While the number of these will vary with the choice of each individual examiner, the following list may be taken as a fair average of what will be required.

### The Instruments Required.

Clinical thermometer, binaural and wooden stethoscopes, steel and linen measures (Wahl's type of steel measure is much used abroad and is excellent), a shoemaker's measure (Langemak also advises the provision of a goniodiameter or Thole and Landwehr's instrument for the measurement of angles, but these are both expensive instruments, and may be dispensed with), ophthalmoscope, laryngeal mirrors with frontal mirror, nasal speculum, aural specula, tongue depressor, œsophageal bougies, small aspirating syringe with needles, apparatus for immediate examination of urine, iron-chloride paper with tannin solution (in wide-mouth glass-stoppered bottle) for taking permanent impressions of flat feet (some authorities recommend a tannin-solution-impregnated felt pad, but the paper is cleaner and much more convenient), ordinary surgical probes, small trocar and cannula, hæmostatic forceps, and scissors.

More expensive diagnostic apparatus such as the bronchoscope, the apparatus for the Wassermann and von Pirquet tests, and that for blood examination, as recommended in Langemak's book, are really unnecessary, as is also the instrumentarium for spinal puncture, since such an operation should not be performed, even for diagnostic purposes, in the examination room. On the other hand a good sphygmomanometer is a valuable aid to diagnosis, which is all too frequently omitted. There are many good modifications of this, but the old Riva-Rocci type serves as well as any other, and may be relied upon. Cases that require more detailed diagnosis, bacteriological or otherwise, should be referred to the in-patient department for observation.

The waiting room should be simply but comfortably furnished, and all apartments should, of course, be suitably warmed and ventilated. As a floor covering linoleum on a cement basis is perhaps the best for these rooms.

### The Rooms and Furniture.

The dressing room is an important adjunct to this division; in it the surgeon and his assistants will deal with such cases as require immediate treatment. It is better not to make it a bandaging room, but to have a separate room for plaster work; but where space cannot be afforded the two rooms must be combined, and can be so combined without loss of efficiency thereby. Dealing with it as a combination of operating and bandaging room, it is essential that it should have a hard non-absorbent flooring: terrazzo, well laid, is the best so far as appearance and durability are concerned, provided a slight slope is afforded so that the floor can be adequately flushed when necessary. Good washing basins, with hot and cold water laid on, must be provided, and in fact the room must be furnished with that due regard to asepsis and surgical cleanliness that should be the rule in every part of the hospital where an operation, no matter how trivial, is carried out. A simple style of adjustable operating table must be included in the fittings, with good head light and well arranged window light. But the most important piece of furniture is the bandaging table. This should be solid and firm. A metal table is not, generally, so convenient and suitable as a heavy wooden one; since "wet" operations are not usually done on this table, the necessity for an impermeable surface is not so urgent. A pelvic rest is a necessity; any type that is firm and simple serves; that of Legal has many advantages. In addition a shoulder support, and padded blocks for use in manipulations of club foot and dislocated hips, must be provided. The table should be fairly narrow and long enough to support an adult comfortably, six feet three by two feet six is a convenient size.

In this room anæsthetising apparatus must be provided, together with an instrumentarium for all minor orthopædic operations; the full list need not be given here. It is of importance to overhaul the instruments carefully every week, and see that they are serviceable and ready for use. A dust tight instrument cupboard, of glass and steel, serves as their resting place when not in use. The next important item in the furnishing is the plaster cupboard which should be of a simple type, with numbered compartments, so that whatever the surgeon wishes may be obtained at a moment's notice without disturbing the other contents of the cupboard. We propose to deal with plaster work in a future article, and need here only say that the various types of plaster bandages, loose plaster, instruments for taking off set plaster bandages, Gigli saws, flexible lead and tin strips, tricot under coverings, wool and lint flannel, and the various other odds and ends that are constantly called into requisition during orthopædic operations, must be represented in the cupboard.

Suspension apparatus, head rests, leg rests, and an osteoclast suggest themselves as additional requirements for this room. Adequate provision must also be made for sterilising instruments, and a special sink must be provided for soaking plaster bandages.

## Figures and Tables

**Figure f1:**